# The Value of Routine Polymerase Chain Reaction Analysis of Intraocular Fluid Specimens in the Diagnosis of Infectious Posterior Uveitis

**DOI:** 10.1155/2013/545149

**Published:** 2013-10-22

**Authors:** Marius A. Scheepers, Karin A. Lecuona, Graeme Rogers, Catey Bunce, Craig Corcoran, Michel Michaelides

**Affiliations:** ^1^Groote Schuur Hospital, Ophthalmology Department, Cape Town, South Africa; ^2^Moorfields Eye Hospital, London, UK

## Abstract

*Objective*. To assess the value of routine polymerase chain reaction (PCR) analysis on intraocular fluid from patients presenting with a first episode of suspected active infectious posterior uveitis in a population with a high prevalence of human immunodeficiency virus infection. *Design*. Retrospective, interventional case series. *Participants*. 159 consecutive patients presenting at a tertiary care hospital over a five-year period. *Methods*. PCR analysis was performed for cytomegalovirus, varicella zoster virus, herpes simplex virus types 1 and 2, *Toxoplasma gondii*, and *Mycobacterium tuberculosis*. *Results*. PCR analysis confirmed the initial clinical diagnosis in 55 patients (35%) and altered the initial clinical diagnosis in 36 patients (23%). The clinical diagnosis prior to PCR testing was nonspecific (uncertain) in 51 patients (32%), with PCR providing a definitive final diagnosis in 20 of these patients (39%); necrotizing herpetic retinopathy and ocular toxoplasmosis were particularly difficult to diagnose correctly without the use of PCR analysis. *Conclusion*. The clinical phenotype alone was unreliable in diagnosing the underlying infectious cause in a quarter of patients in this study. Since the outcome of incorrectly treated infective uveitis can be blinding, PCR analysis of ocular fluids is recommended early in the disease even in resource poor settings.

## 1. Introduction

In developed countries, uveitis affects approximately 200 per 100,000 in the population, and uveitis and its complications account for up to 35% of severe visual impairment [1]. In less developed countries, uveitis and its complications are even more common, affecting an estimated 714 per 100,000 and contributing to 25% of blindness [1]. Posterior uveitis is thought to comprise approximately 5% of all uveitis entities, with the commonest pathogens responsible for *infectious* posterior uveitis and panuveitis being herpes simplex virus (HSV) type 1 and 2, varicella-zoster virus (VZV), cytomegalovirus (CMV), *Toxoplasma gondii* (TG), *Treponema pallidum*, and *Mycobacterium tuberculosis* (MTB) [2]. In developed countries, the most common infectious aetiologies are TG, HSV, and VZV, whereas CMV is a common pathogen in countries with a high prevalence of human immunodeficiency virus (HIV)/acquired immune deficiency syndrome (AIDS) [3–5].

Blindness and visual impairment caused by infectious uveitis can be prevented by early identification of the responsible pathogen and the subsequent prompt administration of appropriate antimicrobial therapy [1]. This is particularly critical in immunocompromised patients [3, 6]. The aetiological diagnosis of infectious uveitis is initially made on the basis of the associated clinical features, but there is often significant overlap between the phenotypic expressions of these different pathogens, thereby limiting the ability to accurately identify the causative organism by clinical examination [7]. Moreover, simultaneous infection of the retina with multiple different organisms in patients with AIDS has been reported, making it almost impossible to make a correct complete diagnosis on clinical grounds alone [8]. Furthermore, establishing a diagnosis based on clinical findings is also difficult in cases where media opacity or poor pupil dilation may mask clinical features. Under these circumstances an incorrect diagnostic decision not only causes a delay of appropriate treatment and prevention of loss of vision but also exposes the patients to side effects of an unnecessary medication [9].

PCR of intraocular fluids is a reliable investigation that can identify most of the common causes of infective posterior uveitis [10]. It is a technique whereby theoretically a single or a few copies of a piece of DNA are amplified across several orders of magnitude, generating millions of copies of a particular nucleic acid sequence. PCR analysis of ocular fluid samples allows accurate and rapid detection of small quantities of DNA or RNA from potential pathogens infecting the eye. It has been shown to be highly sensitive and specific for CMV, HSV, and VZV [2, 6, 9, 11–17]. By comparison, PCR analysis in cases of TG posterior uveitis has a variable sensitivity and a combination of PCR & Goldmann Witmer coefficient analyses improves diagnostic sensitivity [3, 6, 18–25]. PCR diagnosis of ocular MTB has been shown to be highly specific with a variable sensitivity [26]. PCR also offers significantly improved time to diagnosis compared to traditional techniques. 

PCR testing is however not readily available in low income countries and clinicians have to rely on clinical findings to decide on initial treatment. The prevalence of PCR proven causes of infectious uveitis in a population with a high prevalence of HIV/AIDS has not yet been described. This study was therefore performed to determine the prevalence of the commonest causes of infectious uveitis based on PCR studies in a population with a high prevalence of HIV and to document the correlation between the clinical appearance and the laboratory findings in a general ophthalmology clinic to aid the development of suitable treatment protocols.

## 2. Methods

### 2.1. Patients and Clinical Methods

Patients who underwent PCR testing of ocular fluids (vitreous and aqueous) for suspected infectious uveitis at the Ophthalmology Unit in Groote Schuur Hospital between May 1, 2004 and June 30, 2009 were identified. The Ophthalmology Unit at Groote Schuur Hospital in Cape Town, South Africa, is one of 2 tertiary institutions that serve a population of approximately 3 million. The estimated HIV prevalence in this area was 18% in 2009 [27]. Ethics approval for this study was obtained from the University of Cape Town Health Sciences Human Research Ethics Committee.

All patients presenting to the Ophthalmology Triage Division with suspected infectious uveitis underwent routine PCR testing of ocular fluids at the time of presentation. Other investigations included syphilis serology, a full blood count and differential, chest X-ray, and HIV testing if status was unknown. Skin tests for tuberculosis were not commonly performed as it is often of limited value in a population where tuberculosis is endemic.

Based on the phenotypic appearance, the appropriate treatment was commenced pending the results of the PCR testing. When indicated by the subsequent PCR result, treatment was changed. The initial diagnosis and management were made by ophthalmology residents in the Triage Division, after which patients were followed up in the Uveitis Clinic.

The study population consisted of the laboratory sample logs of all patients who underwent PCR testing of ocular fluids between May 1, 2004 and June 30, 2009. Patient charts were reviewed to determine clinical history and course, as well as patient characteristics. Patients who had a known previous episode of posterior uveitis were excluded from the study. PCR testing was performed for the commonest causative organisms, namely, CMV, HSV type 1 and 2, VZV, TG, and MTB.

Ocular fluid samples were obtained by ophthalmology residents and consultants. Vitreous samples were obtained by passing a 23 gauge needle through the pars plana and withdrawing 0.2 to 0.3 mls of core vitreous cavity fluid. A small number of vitreous samples were also obtained at the time of pars plana vitrectomy. If vitreous could not be aspirated, anterior chamber aqueous samples were obtained using a 28 to 30 gauge needle on a 1 mL syringe using tetracaine topical anaesthesia and one drop of topical 5% povidone iodine solution. Samples were then transported urgently to the diagnostic laboratory that was located on the hospital grounds.

## 3. Laboratory Methods for PCR Analysis

### 3.1. Nucleic Acid Isolation

Total nucleic acid was extracted from the aqueous and vitreous fluid using the NucliSens EasyMAG platform (bioMérieux, Boxtel, The Netherlands) according to the manufacturer's instructions. Nucleic acid was eluted in 50 *μ*L elution buffer and stored at −4°C. 

### 3.2. Nested PCR for the Detection of CMV, HSV 1 & 2, VZV, TG, and MTB

In-house nested PCRs were used to screen the samples in this study for CMV, HSV 1 & 2, VZV, TG, and MTB using previously published primer sequences [28–32]. The first round PCR was performed with a 50 *μ*L reaction mixture containing 10 *μ*L extracted DNA, 15 mM Tris-HCL (pH 8), 50 mM KCl, 1.5 mM MgCl_2_, 0.2 mM deoxynucleotide triphosphates (ABgene, Epsom, UK), 20 pmol of each forward and reverse primer, and 1.5 U SuperTherm Taq polymerase (JMR Holdings, Kent, UK). Amplification was performed on a Thermo Hybaid PxE 0.2 thermal cycler (Thermo Scientific, Waltham, MA, USA), with the following conditions: 1 cycle of 94°C for 2 minutes, 40 cycles of 94°C for 20 s, 55°C for 30 s, 72°C for 45 s, and a final elongation step at 72°C for 7 minutes. The second round PCR was performed using the same basic master mix ingredients containing 50 pmol of each inner forward and reverse primer and 2 *μ*L of first round PCR product. Cycling conditions were as for the first round PCR, although the annealing temperature was increased to 58°C. Amplified products were separated by electrophoresis in 2% agarose gel and visualized under UV irradiation after staining with ethidium bromide. The expected sizes of the inner PCR products were 160 bp (CMV), 179 bp (HSV 1 & 2), 251 bp (VZV), 96 bp (TG), and 194 bp (MTB). All work was performed in an ISO-15189 accredited molecular laboratory which employs strict precautions to prevent contamination.

PCR results were reported as detected or not detected within 48 to 72 hours.

## 4. Outcome Measures

Initial pre-PCR diagnoses were made based on history and clinical findings on ocular and systemic examination. Final diagnoses were made based on investigation results, clinical behaviour, and response to treatment. PCR test results were considered to confirm the initial diagnosis if PCR analysis was positive for the pathogen which was considered the inciting cause at presentation. PCR test results were considered to have changed the initial diagnosis if PCR analysis was positive for a different pathogen and the clinical course and response to treatment was consistent with the PCR positive result. If the PCR test results were positive for more than one pathogen and the clinical course and treatment response were consistent with possible coinfection, then the PCR test result was also considered to alter the diagnosis. In all other cases the PCR test result was considered to have an undetermined effect on the final diagnosis. 

There is no gold standard test against which to measure the sensitivity and specificity of PCR analysis in the diagnosis of infectious posterior uveitis [33]. The final diagnoses which were based on the clinical course response to treatment and results of investigations were therefore used as the gold standard in order to calculate an estimate of PCR sensitivity and specificity. The clinical sensitivity and specificity were calculated rather than the nominal sensitivity of the test itself [33]. Estimated positive predictive value (PPV) and negative predictive value (NPV) for PCR testing were also calculated.

Statistical analysis was performed by Dr. C. Bunce from the Moorfields Eye Hospital Medical Statistics Department. A chi-square test was used to compare sensitivity values for PCR testing of vitreous compared to anterior chamber samples.

## 5. Results

Of the 187 consecutive patients who underwent PCR ocular fluid testing, 159 patients were included in the study. There were 28 patients excluded from the study; 11 case notes were damaged or lost, 4 patients had a recurrent episode, 12 patients did not have any active posterior uveitis at the time of sampling, and 1 patient defaulted followup within 1 week of presentation, preventing observation of clinical course and confirmation of the final diagnosis. 

There were no documented complications due to aqueous or vitreous fluid aspiration procedures.

Patient characteristics and average visual acuities pre- and post-treatment are shown in Tables 1 and 2, respectively. The duration of followup ranged from 1 week to 5 years. The number of PCR tests performed for each pathogen tested and the results are shown in Table 3. There were 643 PCR tests performed, with a mean of 4 tests per patient. CMV, VZV, HSV, and TG PCR tests were performed on most patients, whereas MTB PCR was performed less frequently (*n* = 43) as local PCR testing for MTB was only available for the last 18 months of the study (from January 2008). Forty-one patients were tested for all 5 pathogens. 

### 5.1. Initial Clinical Diagnoses

The pre-PCR clinical diagnoses compared with PCR positive findings are shown in Table 4. The most common pre-PCR diagnoses were cytomegalovirus retinitis (CMVR) (*n* = 70), necrotizing herpetic retinopathy (NHR) (*n* = 14), and ocular toxoplasmosis (OT) (*n* = 10). There were 51 patients whose diagnoses were uncertain because their clinical presentations were not characteristic for a particular pathogen. In 17 patients the view of the fundus was so poor due to a combination of severe vitritis and posterior synechiae, that it precluded accurate clinical diagnosis.

### 5.2. PCR Results

Of the 159 patients tested by PCR analysis, 94 patients had a positive PCR result (59%). PCR confirmed the suspected diagnosis in 55 patients (34.6%), altered the diagnosis in 36 patients (22.6%), and had an undetermined effect in 68 patients (42.8%). In the 51 patients who had an uncertain clinical diagnosis, PCR identified 20 patients (39%) with a PCR positive diagnosis consistent with the final diagnosis. CMV PCR tests were the most frequently positive (47%), followed by VZV (11%). There were no positive HSV PCR results.

Five patients tested PCR positive for more than one pathogen. Four patients were CMV and VZV positive, and one patient was CMV and TG positive. In the patients who tested CMV and VZV positive, two were considered to have true active coinfections with CMV and VZV, one had a final diagnosis of CMVR alone, and one had a final diagnosis of NHR due to VZV alone. The patient who tested positive for both CMV and TG had a final diagnosis of OT alone. Three of the five coinfections were therefore considered to be “false positive” results.

Final diagnoses are shown in Table 5. CMV retinitis was bilateral in 36 cases (49%), NHR was bilateral in 6 cases (35%), and OT was bilateral in 2 cases (13%). Using the final diagnoses as the gold standard, estimates of PCR sensitivities for the sampling sites, specificity, positive predictive values, and negative predictive values were calculated and the results are shown in Tables 6 and 7. The overall PCR sensitivity for pathogens tested was 84% (95% confidence interval (CI): 75%–90%). Specificity was 99% (95% confidence interval (CI): 97%–100%). The positive predictive value, defined as the likelihood of having disease related to the tested infectious agent given positive PCR results, was 97% (95% confidence interval (CI): 91%–99%). The negative predictive value, defined as the likelihood of not having the specified disease given negative PCR results, was 95% (95% confidence interval (CI): 92%–97%). 

Despite 148 samples being tested for HSV by PCR analysis, none were HSV positive. Simultaneous vitreous and aqueous specimens were obtained from 4 patients, solitary vitreous samples were taken from 105 patients, and solitary anterior chamber fluid samples from 47 patients. Sampling site information was omitted in 3 case notes (all 3 solitary samples). There were 2 patients who had repeat sampling during their treatment course (both vitreous repeat samples). Overall, vitreous samples had higher sensitivity than aqueous (*P* = 0.027). 

Seventeen patients presented with a very poor view of the fundus. Seven cases were due to OT (4 of these were PCR confirmed) and 2 cases were due to CMVR (both PCR confirmed). Seven cases were idiopathic and one case was due to ocular syphilis.

### 5.3. Bilateral versus Unilateral Disease

Of the 67 patients who presented with bilateral disease, 36 (54%) were due to CMV, 6 (9%) were due to NHR, and 2 (3%) were due to OT. Of the 92 patients who presented with unilateral disease, 38 (41%) were due to CMV, 11 (12%) were due to NHR, and 14 (15%) were due to OT.

## 6. Discussion

This is the first study to describe the pathogen distribution based on PCR testing of patients with infectious posterior uveitis, in a population with a high prevalence of HIV/AIDS. The most frequent final diagnosis was CMVR, followed by NHR and OT (47%, 11%, and 10%, resp.). This is in direct contrast to Harper et al.'s study in a population with a lower HIV prevalence where NHR was the most common diagnosis followed by CMVR and OT [33].

The initial pre-PCR clinical diagnosis was uncertain in 51 cases (32%). This was due to a number of factors. Many patients presented late with significant vitritis and posterior synechiae leading to an obscured fundus view. A significant number of cases presented with atypical findings making it difficult to make a definitive diagnosis. In addition, the initial clinical diagnosis was usually made by general ophthalmologists, not uveitis subspecialists, although this makes the findings of this study more generally clinically relevant, especially in the developing world and also potentially in developed countries. PCR analysis provided the correct final diagnosis in 20 of these patients (39%).

The initial clinical diagnosis changed in approximately a quarter of cases as a result of PCR testing. This is likely due to the significant overlap between the phenotypic expressions of these different pathogens [7]. Having an early definitive laboratory proven diagnosis is advantageous in instituting appropriate effective treatment in a timely fashion; this was not the case in a quarter of our patient population. In our study the clinical diagnosis prior to PCR testing was particularly challenging in patients who were subsequently confirmed to have NHR and OT, often due to a poor view of the fundus. Patients with NHR from VZV infection had findings that overlapped with CMV retinitis, whilst patients with OT were found to overlap with CMV retinitis and NHR phenotypes.

PCR was able to provide a final definitive clinical diagnosis in approximately 60% of our patients. Previous studies have shown PCR analysis of intraocular fluids to detect viral infection in posterior uveitis to be a sensitive and highly specific test. For CMV retinitis sensitivity ranges from 91% to 95% and for NHR sensitivity ranges from 79% to 100% [6, 9, 11–13, 16, 17]. Our study supports these findings with a viral sensitivity of 91% for CMV and 75% for NHR. 

PCR analysis in patients with ocular toxoplasmosis is generally less sensitive than viral retinitis. Studies have shown variable sensitivity ranging from 27% to 85% [6, 18, 21–25]. It has been suggested that in immune compromised patients PCR analysis may have greater sensitivity [6]. This is supported in our study where we found the sensitivity to be 75%. The diagnostic yield for PCR for toxoplasmosis chorioretinitis in this series was comparable with that for viral retinitis (12 of 16 cases, or 75%) and is higher than the 9 of 25 cases (36%) reported by de Groot-Mijnes et al. or by Fardeau et al. [20] for 34 patients with a final diagnosis of toxoplasmosis chorioretinitis, of whom 79% had positive intraocular antibodies and only 27% demonstrated positive PCR results [6, 18]. Fardeau et al. [20] concluded that large lesions in immune compromised individuals were more likely to have positive results. In the series reported by Groot-Mijnes et al., results for intraocular antibody production were positive in 92% of patients, and in contrast to viral retinitis, delayed testing by more than three weeks after onset of symptoms was more likely to lead to positive PCR results for toxoplasmosis. In our study improved sensitivity would most likely have been achieved with the addition of Goldmann Witmer coefficient antibody testing.

The PCR sensitivity for MTB in our study was low. Better PCR tests for MTB are needed. A recent proposal from Gupta suggests that nested PCR may increase the sensitivity but this is not proven in any study with significant numbers [34].

The overall sensitivity of PCR testing in our study was 84% and the specificity was very high at 99%, which are comparable to Harper's study (sensitivity of 81%; specificity of 97%) [33]. The timing of PCR testing may have played a role in the high sensitivity identified in our study; PCR was routinely performed at presentation (when the viral sensitivity is thought to be maximal), as opposed to being used later in the disease if there is no response to initial treatment. 

There were 3 false positive results in our study. Both CMV and VZV were isolated in 2 of these cases, but clinical presentation and course suggested CMV infection only. Both CMV and TG were isolated in the third case, CMV was considered falsely positive as the clinical presentation and course suggested TG infection only. These false positives may have been due to previous resolved infection with a small number of “old” viruses still being present in the eye, or it may be due to virus in the systemic circulation leaking into the eye across a compromised blood ocular barrier, but not causing active infection in the eye. The false negative rate in our study was relatively low. This is in part attributable to the fast transport time to the on-site laboratory and may also be due to the high number of immunocompromised patients in our study. 

The positive predictive value and negative predictive value were both high (PPV = 97%, NPV = 95%). The NPV was high compared to Harper who found their NPV to be 68% [33]. The negative predictive value is a function of both the sensitivity of the test and the prevalence of the disease being tested for. Since PCR testing is particularly sensitive and almost 60% of the study population had infective uveitis, the high negative predictive value is to be expected. This was of particular value in the 51 cases (32%) with uncertain diagnosis, where infectious uveitis could be excluded with confidence following a negative result. 

In our study vitreous samples were more likely to provide a positive diagnosis (*P* = 0.027). There may however been selection bias and as a result it is difficult to draw any definite conclusion from this finding. Harper's study showed better sensitivity for aqueous compared to vitreous samples, but their findings were not statistically significant [33]. No randomized trials exist at present to prove which is better. 

No HSV was detected in any of our patients. Laboratory error was thought to be unlikely as external investigators confirmed the validity of the local laboratory HSV PCR detection method. Also, the local laboratory cerebrospinal fluid HSV PCR detection rate in patients with suspected HSV encephalitis is similar to published studies from around the world. This points to the possibility of a different epidemiology of necrotizing herpetic retinitis in our local population. 

A large proportion of eyes presented with very poor vision. Although there was no improvement in the mean visual acuities of affected eyes, we believe that by instituting the appropriate treatment we may have prevented more eyes from going blind. 

There are a number of inherent limitations to our study. It was retrospective and criteria for performing PCR were not specified, although all patients with presumed infectious uveitis would have had PCR testing undertaken. Some patients had short followup and there was a relatively high dropout rate to followup. Also, this was not a population-based study but a referral centre study resulting in possible selection bias.

In summary, the prevalence of the commonest causes of infectious posterior uveitis based on PCR studies in a population with a high prevalence of HIV was CMV in 47%, VZV in 11%, and OT in 10%. Tuberculosis was rare and HSV was not identified. On the basis of these findings, in the absence of the availability of PCR testing, the treatment of infectious posterior uveitis with intravitreal ganciclovir and systemic acyclovir would be appropriate in 58% of cases. PCR testing changed the diagnosis in a quarter of cases and confirmed the presence of infective uveitis in another third of cases. Since the outcome of incorrectly treated infective uveitis can result in irreversible blindness, PCR analysis of ocular fluids is recommended early in the disease process even in resource poor settings.

## Figures and Tables

**Table 1:** Patient characteristics.

Mean age	34 (range 14–53)
Gender	
Male	58
Female	101
Laterality	
Bilateral	67
Right eye	42
Left eye	50
HIV +ve	142
HAART treatment at presentation	65
MTB treatment at presentation	67

HIV: human immunodeficiency virus.

HAART: highly active antiretroviral treatment.

**Table 2:** Average visual acuities (VA) pre- and post-treatment.

	Pre-treatment	Post-treatment
VA ≥ 6/18	42%	33%
VA 6/24–4/60	16%	14%
VA ≤ 3/60	42%	53%
Eyes assessed	226	191

Thirty-five eyes were lost to followup (due to patients defaulting followup before completion of treatment).

**Table 3:** PCR tests performed.

Infectious agent	Number tests perf.	Number +ve	% +ve
CMV	154	72	47%
TG	150	12	8%
VZV	148	17	11%
HSV	148	0	0%
MTB	43	1	2%

Total tests	643	102	16%

**Table 4:** Pre-PCR clinical diagnoses correlated with PCR positive results.

Pretest diagnoses	Number	CMV	VZV	HSV	TG	MTB	CMV & VZV	CMV & TG	PCR +ve	
		+ve	+ve	+ve	+ve	+ve	+ve	+ve	Rate number	%
CMV	70	51	4		4		1		60	86%
NHR	14	3	2		3		2		10	71%
TG	10	1			1				2	20%
MTB	7					1			1	14%
Syphilis	5								0	0%
IRU	2								0	0%
Unsure	51	11	5		3		1	1	21	41%

Total	159	66	11	0	11	1	4	1	94	59%

IRU: immune reconstitution uveitis.

**Table 5:** Final diagnoses.

CMVR	74
NHR	17
OT	16
MTB	5
Syphilis	4
CMV/VZV coinfection	2
HIV-associated retinopathy	2
Toxocariasis	1
Immune reconstitution uveitis	1
Blood dyscrasia	2
Idiopathic or end-stage late presentation	35

**Table 6:** Estimated sensitivity, specificity, positive predictive value, and negative predictive value for each pathogen tested and broken down into AC and vitreous samples.

	Sample number	True +ve		False +ve		True −ve		False −ve		Sens	Spec	PPV	NPV
CMV	154	69	45%	1	1%	74	48%	7	5%	91%	99%	99%	91%
AC	45	13	29%	0	0%	31	69%	1	2%	93%	100%	100%	97%
V	106	56	53%	1	1%	43	41%	6	6%	90%	98%	98%	88%
NS	3	2	67%	0	0%	1	33%	0	0%	—	—	—	—
VZV	148	15	10%	2	1%	126	85%	5	3%	75%	98%	88%	96%
AC	43	1	2%	0	0%	39	91%	3	7%	25%	100%	100%	93%
V	102	14	14%	2	2%	84	82%	2	2%	88%	98%	88%	98%
NS	3	0	0%	0	0%	3	100%	0	0%	—	—	—	—
TOXO	150	12	8%	0	0%	134	89%	4	3%	75%	100%	100%	97%
AC	45	1	2%	0	0%	43	96%	1	2%	50%	100%	100%	98%
V	102	11	11%	0	0%	88	86%	3	3%	79%	100%	100%	97%
NS	3	0	0%	0	0%	3	100%	0	0%	—	—	—	—
MTB	43	1	2%	0	0%	39	91%	3	7%	25%	100%	100%	93%
AC	20	1	5%	0	0%	16	80%	3	15%	25%	100%	100%	84%
V	22	0	0%	0	0%	22	100%	0	0%	0%	—	—	100%
NS	1	0	0%	0	0%	1	100%	0	0%	—	—	—	—

Total	495	97	20%	3	1%	373	75%	19	4%	84%	99%	97%	95%

AC: anterior chamber; V: vitreous; NS: not specified; Sens: sensitivity; Spec: specificity; PPV: positive predictive value; NPV: negative predictive value.

(1) Three patients had samples taken from an unknown site (not specified in the clinical notes).

(2) Simultaneous vitreous and aqueous sampling was performed on 4 patients.

(3) Two patients had repeat sampling (both were vitreous sample repeats).

**Table 7:** Comparison of estimated sensitivity, specificity, positive predictive value, and negative predictive value for specimen sites.

	Sample number	True +ve		False +ve		True −ve		False −ve		Sens	Spec	PPV	NPV
AC	153	16	10%	0	0%	129	84%	8	5%	67%	100%	100%	94%
V	332	81	24%	3	1%	237	71%	11	3%	88%	99%	96%	96%
NS	10	2	20%	0	0%	8	80%	0	0%	—	—	—	—

Total	495	97	20%	3	1%	373	75%	19	4%	84%	99%	97%	95%

AC: anterior chamber; V: vitreous; NS: not specified; Sens: sensitivity; Spec: specificity; PPV: positive predictive value; NPV: negative predictive value.

(1) Three patients had samples taken from an unknown site (not specified in the clinical notes).

(2) Simultaneous vitreous and aqueous sampling was performed on 4 patients.

(3) Two patients had repeat sampling (both were vitreous sample repeats).
